# Building an antimicrobial stewardship program: A narrative of six years under the Donabedian perspective

**DOI:** 10.3389/fphar.2023.1074389

**Published:** 2023-03-21

**Authors:** Amanda Fonseca Medeiros, Caryne Margotto Bertollo, Adriano Max Moreira Reis, Monica Aparecida Costa, Edna Marilea Meireles Leite, Simony da Silva Gonçalves, Mauro Henrique Nogueira Guimarães de Abreu, Renan Pedra de Souza, Maria Auxiliadora Parreiras Martins

**Affiliations:** ^1^ Faculdade de Farmácia, Universidade Federal de Minas Gerais, Belo Horizonte, Minas Gerais, Brazil; ^2^ Hospital Risoleta Tolentino Neves, Rua das Gabirobas, Belo Horizonte, Minas Gerais, Brazil; ^3^ Hospital das Clínicas da Universidade Federal de Minas Gerais, Belo Horizonte, Minas Gerais, Brazil; ^4^ Faculdade de Odontologia da Universidade Federal de Minas Gerais, Belo Horizonte, Minas Gerais, Brazil; ^5^ Departamento de Genética, Ecologia e Evolução, Instituto de Ciências Biológicas da Universidade Federal de Minas Gerais, Belo Horizonte, Minas Gerais, Brazil; ^6^ Faculdade de Medicina da Universidade Federal de Minas Gerais, Belo Horizonte, Minas Gerais, Brazil

**Keywords:** Antimicrobial Stewardship, drug resistance microbial, outcome assessment health care, outcome and process assessment, health care, quality assurance, health care, pharmacoepidemiology

## Abstract

**Introduction:** Antimicrobial resistance (AMR) is increasing and represents one of the world’s major challenges. AMR increase morbimortality, length of hospital stay and costs. Antimicrobial Stewardship Programs (ASP) are one of the key strategies to promote the rational use of antimicrobials since AMR is mostly driven by antimicrobial consumption.

**Objective:** To describe the ASP implementation in a teaching hospital from the perspective of Donabedian quality assessment and the Brazilian regulatory requirements.

**Method:** This was a descriptive study with secondary data collection, including document review of the ASP. The study setting was a general public 392-bed hospital. The ASP activities were performed by the hospital infection control committee (HICC), hospital pharmacy (HP) and diagnostic support laboratory (DSL). The description of the three services mainly involved in the ASP was based on a quality assessment model involving the dimensions of “structure”, “process” and “result” proposed by Donabedian. The distribution among dimensions was guided by the checklist of essential elements of the ASP that compose the Brazilian regulatory requirements. The checklist was applied in July, 2022, and the ASP results described from 2016 to 2021.

**Results:** ASP actions have been gradually implemented since 2008 with the implementation of HICC and improved over the years. Regarding structure, the investments in technology were mapped, quantifying 26 computers and three software programs employed to computerize the ASP processes performed in specific physical areas by HICC, HP and DSL. Institutional guidelines used by HICC, HP and DSL guided clinical practices to operationalize ASP. The evaluation metrics improved for 10 indicators and worsened for four indicators. From the 60 items composing the checklist, the hospital met the requirements in 73.3% of the items (n = 44).

**Conclusion:** This study described the implementation of ASP in a teaching hospital, applying the Donabedian perspective. Although the hospital still does not have a classic ASP model, there were investments to improve structure, processes and results, aiming to comply with international guidelines. A high proportion of key elements of ASP in the hospital were followed according to the Brazilian regulatory requirements. Aspects related to antimicrobial consumption and the emergence of microbial resistance deserve further investigations.

## Introduction

Antimicrobial resistance (AMR) is one of the world’s major challenges in terms of global health, food safety, environmental wellbeing and socio-economic development, presenting a gradual increase on all continents (WHO, 2019). AMR is considered a major public health threat contributing to elevated morbidity, mortality, prolonged hospital stays and significantly increased hospital costs (O’Riordan et al., 2021; Vicentini et al., 2023).

Inappropriate or excessive use of antimicrobials are important determinants of AMR due to the ecological impact of these agents (Vicentini et al., 2023). Previous estimates reported that 25%–50% of hospitalized patients use antibiotics, of which 20%–50% are unnecessary or inappropriate (Kallen et al., 2019; van den Bosch et al., 2016). The pace of development of new antimicrobials is slower than the global demand (WHO, 2017; WHO, 2021). Therefore, the development of strategies to promote appropriate use of antimicrobials is essential to reduce the selective pressure for emergence of AMR and to improve patient safety (Vicentini et al., 2023).

As a result of the growth of AMR, multiple international and national initiatives have been implemented to increase the appropriate use of antimicrobials and reduce AMR (da Silva et al., 2021; Godman et al., 2021). Antimicrobial Stewardship Program (ASP) has been reported as an effective strategy to optimize the use of antimicrobials in hospitals (Vicentini et al., 2023). ASP comprises a coherent set of collective actions, developed daily, aiming to: 1) Promote responsible use of antibiotics; 2) Achieve treatment effectiveness; 3) Reduce the likelihood of infections and; 4) Minimize adverse events, including AMR (Dyar et al., 2017; Vicentini et al., 2023).

World Health Organization (WHO) is leading the process of further development and consolidation of a functional global surveillance system on antimicrobial usage and its consequences. This system will be able to operate in diverse economic and socio-political contexts, and still provide timely and reliable data. Pharmacoepidemiological studies have been designed to determine the pattern of antimicrobial drug use in hospitals and contribute to this local, national, and international surveillance system.

The Brazilian Health Regulatory Agency (Anvisa) published in 2017 the National Plan for Antimicrobial Resistance Prevention and Control in Health Services. This is a reference to guide strategies and actions consider focused on the detection, prevention and control of the dissemination of resistant microorganisms by employing a systematic and fast approach, based on scientific and laboratory evidence (Brazilian Health Regulatory Agency, 2017a). Afterwards, the National Guidelines for the Management Program of Antimicrobial Use in Health Services was published, encouraging the implementation of ASP in Brazilian hospitals (Brazilian Health Regulatory Agency, 2017b). The guideline was developed to comprehensively present the key elements of an ASP that should be adapted according to reality, local needs, epidemiological and microbiological profiles, barriers and hospital resources aiming to optimize the use of antimicrobials (Brazilian Health Regulatory Agency, 2017b).

Most studies describing the process of ASP implementation in hospitals and the assessment of its performance over time have been developed in high-income countries (Cox et al., 2017). There are few studies discussing the experience of ASP implementation in low- and middle-income countries, such as Brazil (Bizerra, 2020; Sato et al., 2021; Menezes et al., 2022), as well as the methods for program evaluation and feedback to healthcare teams and hospital leadership. Thus, the aim of this study was to describe the ASP implementation in a teaching hospital from the Donabedian perspective and the Brazilian regulatory requirements, assessing the structure, work processes and the evaluation metrics monitored over six years.

## Material and methods

This was a descriptive study with secondary data collection, including document review of the ASP and evaluation metrics obtained from the actions of infection control. The study setting was a general public teaching hospital which belongs to the Brazilian Unified Health System. This 392-bed hospital is a referral center for more than 1.2 million people living in the metropolitan region of Belo Horizonte, Minas Gerais State, in the Southeast Brazil. The hospital provides care for about 50 thousand patients per year, encompassing medium and high complexity assistance for clinical, surgical and polytrauma emergencies, as well as maternal and child care. The study protocol was approved by the Institutional Ethics Committee of the Universidade Federal de Minas Gerais (CAAE 54060321.8.0000.5149). Informed consent form was waived due to the aggregated analyses of data.

**FIGURE 1:**
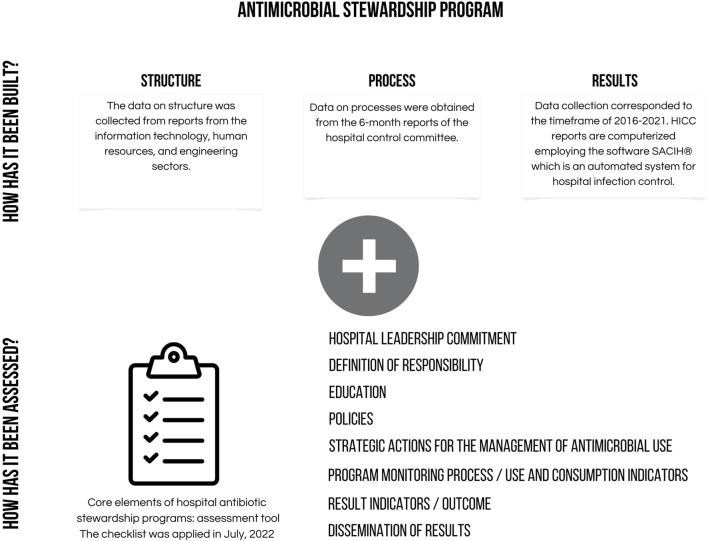
Schematic approach to evaluation of Antimicrobial Stewardship Programme.

The ASP activities of the study hospital are guided by the hospital infection control committee (HICC), hospital pharmacy (HP) and diagnostic support laboratory (DSL). The description of the three main services involved in the ASP was based on a quality assessment model involving the dimensions of “structure”, “process” and “result”, according to the triad proposed by Donabedian (Donabedian, 1990) (Figure 1). The distribution among dimensions in the quality assessment was guided by the checklist of essential elements of the Hospital Antimicrobial Use Management Program, adapted by Anvisa from the Core Elements of Hospital Antibiotic Stewardship Programs proposed by the Center for Disease Control (CDC). Anvisa is responsible for infection control and monitoring drug usage in hospitals and community in Brazil (Brazilian Health Regulatory Agency, 2017b) (Figure 1). The checklist applied comprised eight sections with specific items with “yes/no” questions. The sections consist of: 1) support from the hospital senior management; 2) definition of responsibilities of professional teams; 3) educational activities; 4) actions to improve antimicrobial prescribing; 5) monitoring of the program; 6) results/outcomes; and 7) dissemination of results. For the purpose of the present study, the checklist was applied in July, 2022. A flowchart of the process of use of antimicrobials was also provided.

The dimension of “results” was extracted from the HICC reports that follow the standard of the National Healthcare Safety Network (NHSN) (Dudeck et al., 2013) protocols. Data collection corresponded to the timeframe of 2016–2021. HICC reports are computerized employing the software SACIH^®^ which is an automated system for hospital infection control, covering the following metrics: risk of health care-associated infection, risk of surgical site infection, risk of post-surgical infection and risk of infection in the intensive care unit (ICU). The incidence densities were described for: Catheter-associated urinary tract infection in the ICU; pneumonia associated with mechanical ventilation in the ICU; primary sepsis associated with central venous catheter in the ICU; vascular access infection associated with central venous catheter in the ICU; infection caused by species of *Acinetobacter baumannii*; Infection caused by species of *Pseudomonas aeruginosa*; Infection caused by methicillin-resistant *Staphylococcus aureus* (MRSA); Infection in the general internal medicine; Infection caused by bacteria of the group CESP that includes *Citrobacter, Enterobacter, Serratia, Providencia* and *Proteus*; And infection caused by carbapenem-resistant *Klebsiella pneumoniae* (KPC). Data are compiled from HICC, HP and DSL under the supervision of the former and used for metrics’ calculation, employing the SACIH^®^ software. The results were presented by year of extraction and the variation encountered for the period of study was estimated from the time series, subtracting the value obtained in 2021 by that of 2016. Data on structure was collected from reports available at the departments of information technology, human resources, and engineering. Data on processes were obtained from each of the 6-month reports of the HICC. The results described from the Donabedian perspective and applying the 60-item Anvisa checklist were presented in figures.

## Results

The three hospital services directly involved with ASP (HICC, HP and DSL) are hierarchically linked to the senior leadership. There is no precise date that marks the beginning of ASP in the institution. The policies and actions have been gradually implemented since 2008 with the implementation of the HICC, and improved over the years. All procedures followed the Brazilian regulatory requirements and ASP results were evaluated every six months by a multi-professional committee. The team members directly responsible for ASP have their responsibilities pre-defined in the description of their institutional positions. The hiring process is performed by public selection resulting in reduced turnover and, consequently, minimized impact on the communication among professionals and information management.

Considering the Donabedian triad, the quality assessment of the domains “structure”, “process” and “results” was presented for each service involved in ASP (Supplementary material I). Starting with HICC, the department counted on 13 professionals during all the study period (2016–2021). The HICC performed epidemiological surveillance applying the NHSN/CDC methodology in a systematic, active and continuous form. Inpatients were monitored for health care-associated infections in all topographies and notifications were performed following the diagnostic criteria established by Anvisa (Brazilian Health Regulatory Agency, 2017c). All recommendations for precaution and/or treatment of infections were recorded by HICC in the computerized system to be checked and followed-up by the healthcare team. These data could also be compiled as indicators for assessing the performance of the service on a monthly basis. The results were presented every six months to other healthcare professionals involved in the process.

**FIGURE 2:**
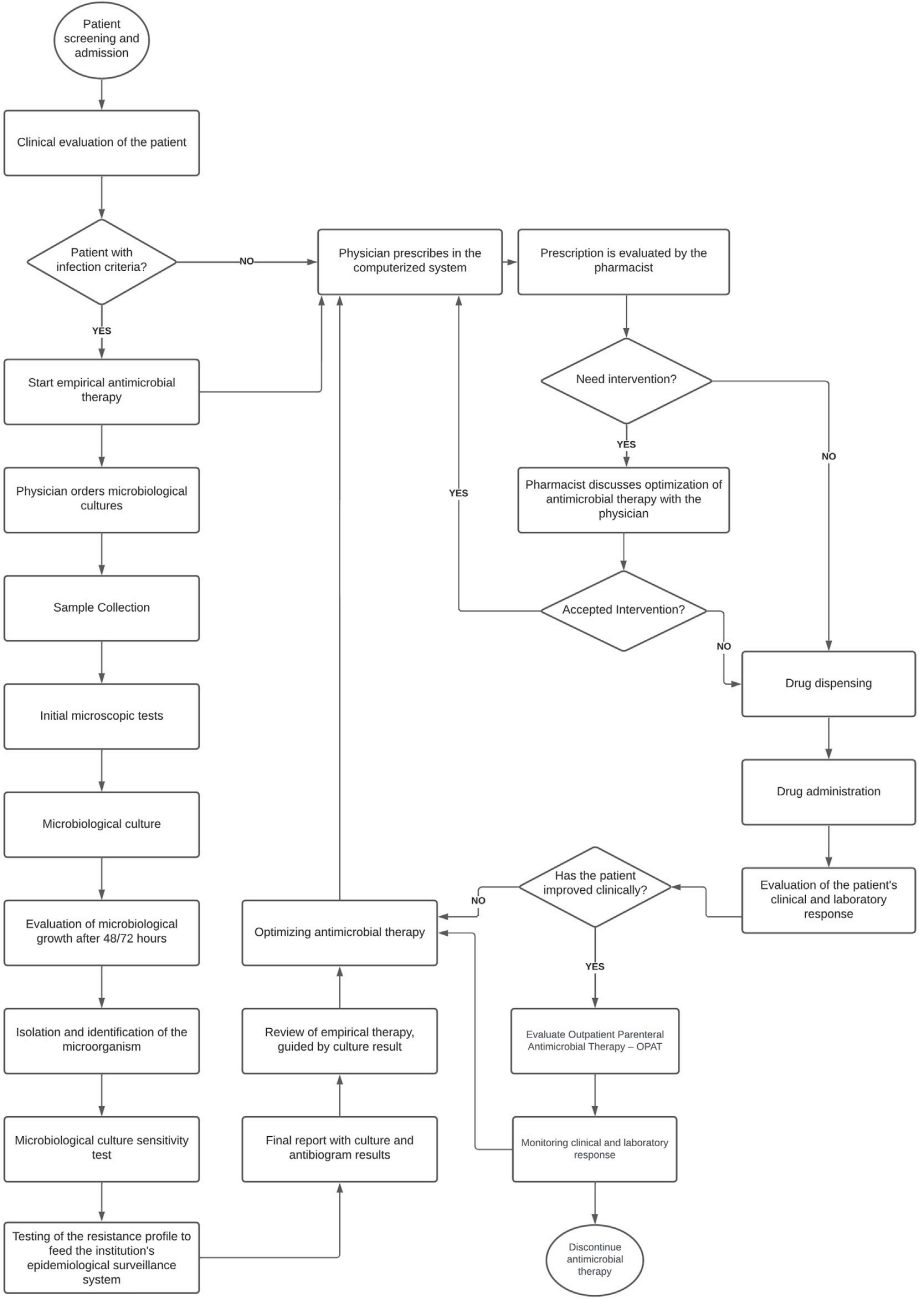
Flowchart of the process of antimicrobial use.

The HP received investments in human resources increasing the number of pharmacists from nine in 2016 to 15 in 2021, and the total of other professionals in the HP from 82 in 2016 to 110 in 2021. Besides, HP activities were expanded in 2016 with the restructuring of clinical pharmacy. The service operated 24 h a day in a physical area distributed into a central pharmacy and six satellite units. Medication prescription was developed by using a Computerized Physician Order Entry (CPOE). Antimicrobials were dispensed according to medical prescription and the analysis of drug indication. The first dose of restricted use antimicrobials was released immediately after the prescription. Then, the pharmacist performed a detailed evaluation of the indication, safety, effectiveness and convenience to substantiate the decision for completing drug therapy. There was no referral pharmacist specifically assigned to the management of antimicrobials and the release of restricted use antimicrobials could be discussed with HICC by any professional on duty. Figure 2 shows the flowchart of antimicrobial use starting from the assessment of clinical condition and the decision for prescription of antimicrobials, or not, until the completing of antimicrobial therapy.

**FIGURE 3:**
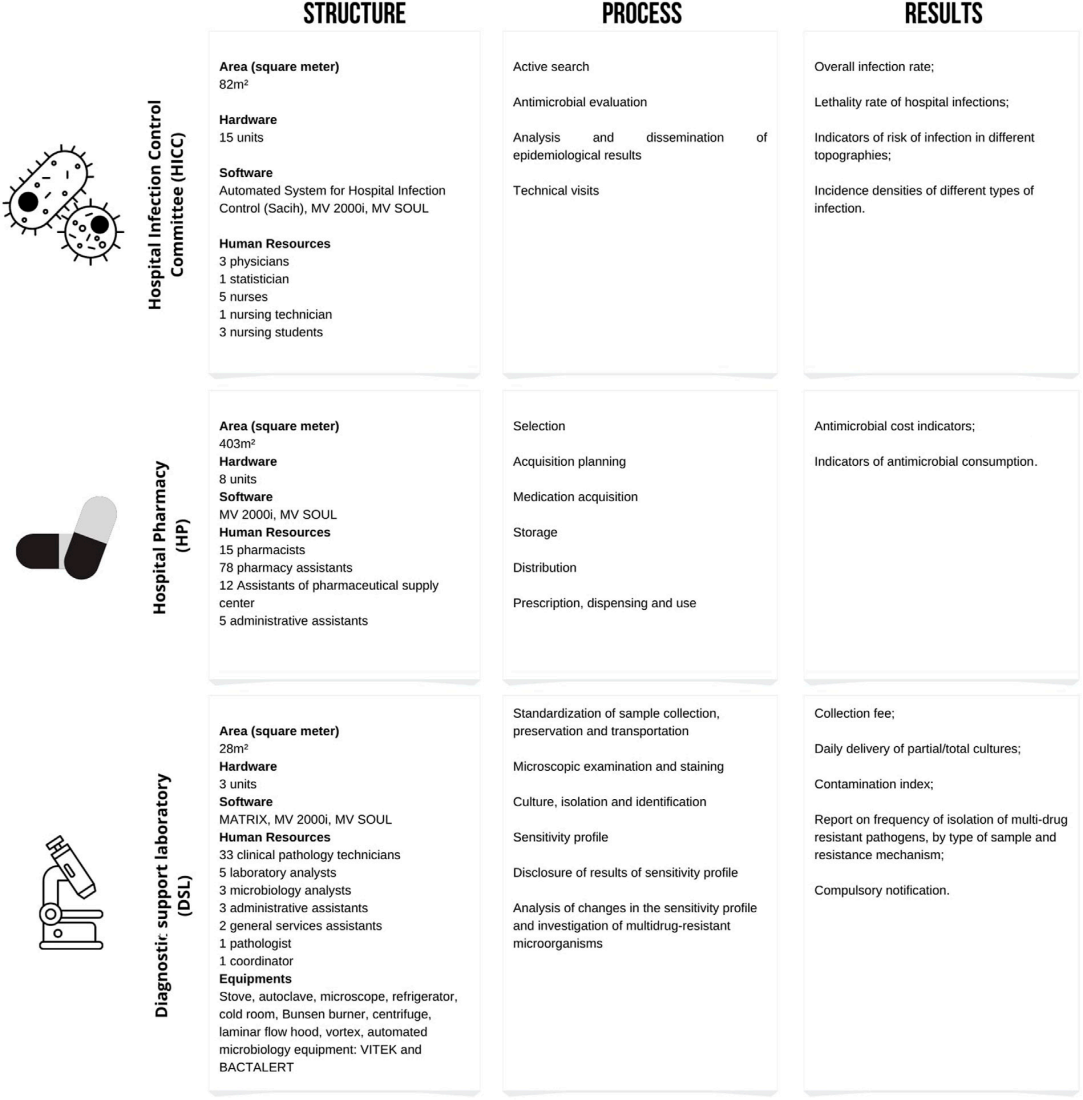
Results of the evaluation from the Donabedian perspective.

The LDS had 47 professionals in 2016 and 48 in 2021, with an additional microbiology analyst. The laboratory belongs to the hospital structure and could perform tests in the fields of microbiology, biochemistry, haematology and others required for special conditions. The processes were developed in a modern technological platform, with traceable information throughout the entire diagnostic chain, from the pre-analysis to final results. The data could be integrated through an interface between the laboratory computer system and the hospital management software, ensuring bidirectional connection of information and greater efficiency in decision making. The collection of biological material for microbiological tests was guided by the infection suspected and developed according to the institutional protocols to ensure quality of tests. Upon receiving the material, the process chain was started including: Microscopy, culture, isolation and identification of pathogens, as well as determination of their sensitivity profile. The results of the evaluation from the Donabedian perspective were summarized in Figure 3.

**FIGURE 4:**
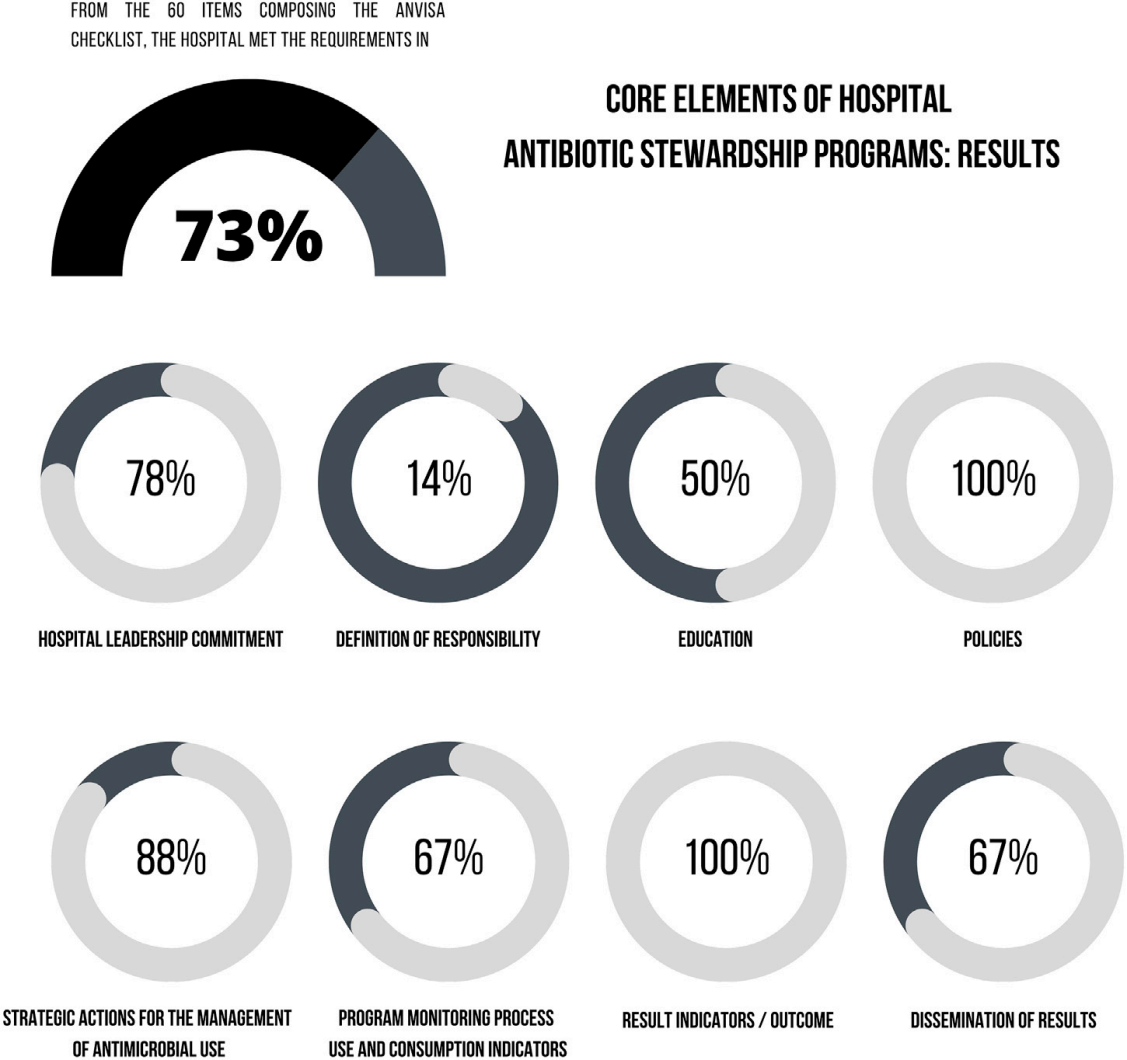
Results for core elements of the Hospital Antibiotic Stewardship Program according to the Anvisa checklist.

From the 60 items composing the Anvisa checklist, the hospital met the requirements in 73.3% of the items (n = 44). The highest proportion of compliance referred to the development of actions to improve antimicrobial prescribing (89.3%; n = 25/28) and to the support from senior management (77.8%; n = 7/9). The results of the checklist application were summarized in Figure 4.

The subsections of Anvisa checklist presenting some level of inadequacy were: 1) Support from the hospital senior management in regard to the availability of departments to contribute to the management of antimicrobial use, as well as the staffing to operate ASP; 2) Definition of responsibilities of professional teams; 3) Educational activities since the hospital did not offer educational programs for patients, companions/caregivers on the correct use of antimicrobials; 4) Actions to improve antimicrobial prescribing in regard to the use of pharmacokinetics/pharmacodynamics knowledge to optimize antimicrobial dosing regimens to treat infection caused by microorganisms with reduced sensitivity, absence of automatic stop orders time-sensitive for specific prescriptions of antimicrobials, especially those administered for surgical prophylaxis, and use of biomarkers for the management of antimicrobial use; 5) Monitoring of the program with the incorporation of indicators of antimicrobial consumption, such as measures of length of therapy (LOT) and days of therapy (DOT).

The results for indicators measured in the study period (2016–2021) are depicted in Table 1. There was a positive variation for 10 indicators and a negative variation for four indicators. The indicators with worse results were risk of post cesarean section infection, risk of infection in the intensive care unit, density of incidence of infection in the general internal medicine and density of incidence of infection caused by KPC.

**TABLE 1:** Main results for metrics regarding actions of hospital infection control.

Indicador	2016	2017	2018	2019	2020	2021	Δ^b^
Risk of health care-associated infection	3,2	2,1	1,9	1,6	1,6	1,8	−1,4
Risk of surgical site infection	5,6	2,8	4,1	4,8	3,3	3,3	−2,3
Risk of post-cesarean section infection	2,3	1,1	1,1	2,6	1,2	3,5	1,2
Risk of infection in the ICU	18	12	26	18	12	22	4
Density of incidence of infection catheter-associated urinary tract infection in the ICU	4,3	4,3	3,3	3,5	1,6	2,5	−1,8
Density of incidence of pneumonia associated with mechanical ventilation in the ICU	23,7	10,9	13,1	6	15,1	15,7	−8
Density of incidence of primary sepsis associated with central venous catheter in the ICU	11,8	7,3	4,2	2,9	3,2	5	−6,8
Density of incidence of vascular access infection associated with central venous catheter in the ICU	1,7	1,1	1,1	1,3	1,4	0,8	−0,9
Density of incidence of infection caused by *Acinetobacter baumannii*	1,4	0,7	0,6	0,7	0,8	1,3	−0,1
Density of incidence of infection caused by *Pseudomonas aeruginosa*	1,1	0,8	0,6	0,7	0,9	0,6	−0,5
Density of incidence of infection caused by methicillin-resistant *Staphylococcus aureus* (MRSA)	0,6	0,3	0,2	0,2	0,3	0,5	−0,1
Density of incidence of infection in the general internal medicine	7,9	6,6	6,9	7,2	4,9	8,6	0,7
Density of incidence of infection caused by bacterias of the do group CESP^a^	1,2	1,3	1,5	1,1	1,3	0,8	−0,4
Density of incidence of infection caused by *Klebsiella pneumoniae* carbapenem-resistant (KPC)	0,3	0,5	0,8	0,6	0,7	0,8	0,5

^a^ CESP—*Citrobacter*, *Enterobacter*, *Serratia*, Providencia, *Proteus*.

^b^ Delta coefficient (Δ) was calculated by the difference of the value obtained in 2021 minus the value in 2016. This table displays the annual estimates and does not constitute a statistical trend analysis.

## Discussion

The findings showed that the studied hospital still does not have a classic ASP model, but presented improvements in structure, processes and results. A high proportion of key elements of ASP were followed in accordance with the Brazilian regulatory requirements. ASP is a complex intervention that relies on the interaction between multiple actors (Hughes et al., 2022).

The dimension of “structure” involves the organization and requirements of the multidisciplinary team providing means to implement and support the multifaceted activities of an ASP (O’Riordan et al., 2021). The use of antimicrobials in a system with adequate informational support was a positive factor, showing greater traceability and control along the chain. The study hospital lacked a specific operational team responsible for ASP implementation and development. This aspect has been reported as the main barrier for ASP implementation in adult ICUs in Brazil, according to a survey developed by Anvisa in 2019 (Brazilian Health Regulatory Agency, 2019). To obtain better results, the guidelines recommend that ASP should be developed by a specialized team (Barlam et al., 2016; Brazilian Health Regulatory Agency, 2017b; Doernberg et al., 2018). In general, HICC and HP develop accumulate activities involving ASP and other different tasks in their daily routine. The ideal team would be multi-professional, including an infectious disease specialist, a clinical pharmacist trained in infectious diseases, and a clinical microbiologist (Dellit et al., 2007).

The support of the hospital leadership is a structuring element to enhance local results (Dellit et al., 2007; Brazilian Health Regulatory Agency, 2017b; Brazilian Health Regulatory Agency, 2021) by formally assuming a commitment with ASP through a policy that provides funding, clear definition of responsibilities and sufficient human resources, that are in line with the international recommendations (Brazilian Health Regulatory Agency, 2021). Despite the importance of this element, in regulatory terms, the hospital complies with the current requirements of Anvisa which are designed in an intermediate level (Ministry of Health, 1998; Brazilian Health Regulatory Agency, 2017b; Brazilian Health Regulatory Agency, 2021). Brazil still has a long way to implement broader and more efficient ASP actions in hospitals.

The dimension “process” involving healthcare teams was mostly characterized by the fragmentation of the activities performed and by the way of communication with prescribers on actions to improve the prescription of antimicrobials that occurred *via* instant messaging applicative and/or records in medical charts. The audit of the use of antimicrobials should be prospective, but this recommendation may not have been followed due to the lack of exclusive professionals and a specific operational team assigned for this function. Thus, the detection of a necessary intervention and the feedback to the attending physician may be delayed.

Education is an essential element of ASP, but passive educational strategies (lectures, events, booklets) without active interventions have shown to be little effective for changing the practices of antimicrobial prescribing and their impact is not sustained over time (Dellit et al., 2007; Barlam et al., 2016). The study hospital offered continuing education using active methodologies focused on raising awareness of professionals on the need to promote appropriate use of antimicrobials and to prevent multidrug resistance. This education program included physician training on good practices of antimicrobial prescribing. The teaching role was extended to undergraduate and graduate students by integrating hospital practices with the curricula covering topics on the rational use of antimicrobials (Donabedian, 1990; Brazilian Health Regulatory Agency, 2017b; O’Riordan et al., 2021). Future perspectives comprise the need to expand education programs to empower patients and companions/caregivers and to improve patients’ self-care.

Protocols were widely disseminated in the hospital and kept available for clinical staff, covering the main infectious diseases requiring antimicrobial use. The implementation of guidelines can be facilitated by educational interventions with feedback on the use of antimicrobials and patient outcomes (Gyssens, 2018). The effect of training should be measured and assessed according to the quality of antimicrobial prescribing, the expansion of microbial resistance and other indicators recommended by Anvisa (Barlam et al., 2016; Brazilian Health Regulatory Agency, 2017b).

The Infection Disease Society of America (IDSA) suggested that computerized clinical decision support can facilitate the procedures of ASP (Barlam et al., 2016). The computer system integrates CPOE and clinical data in the study hospital. However, the functions of the computer system and the lack of data input on medication administration do not allow issuing automatic orders to stop specific antimicrobial prescriptions, especially those involving surgical prophylaxis, representing a process to be improved.

Interventions in the dimension of “process” are considered as part of the most effective ASP strategies to improve antimicrobial prescribing in hospitals (Davey et al., 2017; O’Riordan et al., 2021). Economic assessment should be performed before the implementation or adaptation of prescribing practices (Nathwani et al., 2019). Pharmacokinetic and pharmacodynamics modeling has been reported as a useful strategy to provide a more rational individualization of antimicrobial dosing regimens, increasing the effectiveness of infection treatment. However, modeling strategies are not widely available in this hospital due to the lack of specialized resources, such as specific software and qualified staff, as reported in the literature (Owens et al., 2018).

Regarding the dimension of “results”, the hospital monitored the consumption of antimicrobials through the defined daily dose (DDD) recommended by WHO (Brazilian Health Regulatory Agency, 2017b; WHO, 2023). This measure has been mostly used due to the simplicity of data collection in the computer systems. Although the measure of days of therapy (DOT) is considered more accurate, DDD remains as a feasible alternative for institutions with limitations in collecting data per patient (Barlam et al., 2016). The assessment of these metrics (DDD or DOT) is highly dependent on the availability of CPOE and electronic records of medication administration to allow data collection at the patient level (Kallen et al., 2019). This process should be improved in the study hospital and deserves to be investigated in future studies.

Among the metrics with worsening results, there was an increase in the risk of post-cesarean section infection, risk of infection in the ICU, density of incidence of infection in the general internal medicine and density of incidence of infection caused by KPC. These metrics may have been influenced by the COVID-19 pandemic that caused a substantial overload to healthcare systems with hospital overcrowding, overload of teams, continuous and rapid changes in hospital practices, increased length of hospital stay, greater exposure to invasive devices, and shortage of drugs, materials and medical equipment (Lastinger et al., 2022). In the hospital under study, the KPC was a microorganism recognized as one of the main institutional problems, a finding in line with the WHO report that identifies it as a pathogen of global priority due to the high mortality risk associated, high rate of transmissibility, presenting a reduced treatment arsenal and major repercussions for health services and the community (WHO, 2017; WHO, 2021).

Prescribers receive direct and personalized feedback on ASP results with suggestions on procedures to enhance the quality of their prescriptions of antimicrobials. Even so, the study hospital recognizes this communication can be improved. The dissemination of ASP results was done twice a year in meetings involving HICC members and hospital leaderships, but the information was not systematically disseminated to all hospital workers. Future perspectives include the need to expand the process of continuous communication with healthcare professionals aiming to promote their engagement and the development of ASP actions in clinical practice (Dellit et al., 2007; Barlam et al., 2016; Brazilian Health Regulatory Agency, 2017b; Dyar et al., 2017; Doernberg et al., 2018; Luther et al., 2018; Bleasdale et al., 2019; Brazilian Health Regulatory Agency, 2021).

The applicability of this study occurs through the possibility of mapping the reality of structure, working processes and results of ASP in Brazil and bringing potentially useful information for planning ASP implementation in other middle-income countries. The employment of quality assessment in the healthcare environment for ASP was a potential strength of this research, along with the discussion of the complexity of this practice adapted to a real micropolitical context. This study allowed obtaining a comprehensive overview of the use of antimicrobials, incorporating a multifactorial view. Moreover, in the scope of pharmacoepidemiology, drug utilization studies are important to deepen the assessment of the performance of ASP on the effectiveness and safety of the use of antimicrobials.

There are some limitations to be addressed. Potential information bias may involve subjectivities related to the hospital self-assessment on the compliance with the essential elements listed for ASPs. There were no modifications in the laboratory diagnosis methods or any work processes regarding practices for infection control, although the implementation of other quality improvement projects at the same time of ASP could confound the measurement of outcomes. The implementation of the patient safety program occurred during the study period and may have influenced the ASP outcomes, but potential confounders were not measured in this study. Further studies should be developed to investigate the relationship between antimicrobial consumption and the emergence of resistance employing time series, bringing contributions to the decision-making process by the hospital leaderships on ASP actions.

## Conclusion

This study described the implementation of ASP in a teaching hospital, applying the Donabedian perspective. Although the hospital still does not have a classic ASP model, there were investments to improve structure, processes and results, aiming to comply with the international ASP guidelines. There was a high proportion of key elements of ASP in the hospital, following the national regulatory requirements and showing efforts to strengthen ASP implementation in the hospital. Further studies should be developed to investigate aspects related to antimicrobial consumption and the emergence of microbial resistance.

## Data Availability

The original contributions presented in the study are included in the article/Supplementary Material, further inquiries can be directed to the corresponding author.
