# Supplementary material for: Building an antimicrobial stewardship program: A narrative of six years under the Donabedian perspective
Source: Front Pharmacol. 2023 Mar 21;14:1074389. doi: 10.3389/fphar.2023.1074389 (PMC10070795; doi:10.3389/fphar.2023.1074389)
Supplement: Supplementary file 1 [file DataSheet1.pdf]

## SUPPLEMENTARY MATERIAL

## I - Donabedian Triad of structure x processes x results in the last year of the study

| Hospital Infection Control Committee (HICC) | | |
|---|---|---|
| STRUCTURE | PROCESS | RESULT |
| **Area (square meter)**<br>82$m^2$<br>**Computers**<br>15 units<br>***Software***<br>Automated System for Hospital Infection Control (Sacih®), MV 2000i, MV SOUL<br>**Human Resources ***<br>3 physicians<br>1 statistician<br>5 nurses<br>1 nursing technician<br>3 nursing students | **Active search:** analysis of all medical records taking into consideration the following epidemiological clues: culture results, antibiotic therapy, radiological exams and signs and symptoms described by the physician. After discharge, surveillance of egresses: carried out by phone call, message via WhatsApp and return to the outpatient clinic, when over 90% of patients were reassessed by the physician.<br><br>**Antimicrobial evaluation:** performed by the HICC physician, daily, in all patients taking therapeutic or prophylactic antibiotics.<br><br>**Analysis and dissemination of epidemiological results:** reports are carried out with analysis by the HICC and discussion with clinical staff and management every six months.<br><br>**Technical visits:** carried out in all hospital departments aiming to verifying if patient care processes are in accordance with the HICC protocols. The solution of non-conformities was discussed with the Quality Management Centre and the sectors involved. | Overall infection rate;<br><br>Lethality rate of hospital infections;<br><br>Indicators of risk of infection in different topographies;<br><br>Incidence densities of different types of infection. |
| **Hospital Pharmacy (HP)** | | |
| **Area (square meter)**<br>403$m^2$<br>**Computers**<br>8 units<br>***Software***<br>MV 2000, MV SOUL<br>**Human Resources***<br>15 pharmacists<br>78 pharmacy assistants | **Selection:** the pharmacist participates in the process of drug selection, including antimicrobials, assessing cost-effectiveness and safety. The Pharmacy and Therapeutics Committee is coordinated by a pharmacist who conducts the process together with a multi-professional team.<br><br>**Acquisition planning:** carried out | Antimicrobial cost indicators;<br><br>Indicators of antimicrobial consumption; |

| | | |
|---|---|---|
| 12 Assistants of pharmaceutical supply center<br>5 administrative assistants | annually by a pharmacist, based on consumption and seasonality reports.<br><br>**Medication acquisition:** carried out by a purchasing department. Technical analyses of documents and drug reports are carried out by a pharmacist.<br><br>**Storage:** performed by a pharmaceutical supply center, following good medication storage practices. The process is coordinated by a pharmacist who gives guidelines to the middle-level team. There are no pharmacy technicians in this unit. All product fractioning and unitization processes are performed at this step.<br><br>**Distribution:** this step is done by the middle level team, under the supervision of a pharmacist, based on the average consumption in the department. Satellite pharmacies are resupplied on a daily basis.<br><br>**Prescription, dispensing and use:** medication is dispensed in a mixed manner - oral liquid medications not subject to special control are dispensed to departments, and the other medications are dispensed individually to the patient, upon presentation of the computerized medical prescription. The restricted use antimicrobials undergo additional control, being released only after approval by the HICC and analysis by the pharmacist. | |
| **Diagnostic support laboratory (DSL)** | | |
| **Area (square meter)**<br>28m²<br><br>**Computers**<br>3 units<br><br>***Softwares***<br>MATRIX, MV 2000i, MV SOUL<br><br>**Equipaments**<br>Stove, autoclave, microscope, refrigerator, cold room, Bunsen burner, centrifuge, laminar flow | **Standardization of sample collection, preservation and transportation:** the collection of biological material is done by the nursing team or by the medical team depending on the target anatomical site. The guidelines for preparation, collection and transportation of the sample to the laboratory are made by the microbiology team.<br><br>**Microscopic examination and staining:** direct tests may be | Collection fee;<br>Daily delivery of partial/total cultures;<br>Contamination index;<br>Report on frequency of isolation of multi-drug resistant pathogens, by type of sample and resistance mechanism;<br>Compulsory notification. |

| | | |
|---|---|---|
| hood, vortex, automated microbiology equipment: VITEK and BACTALERT | performed without staining, Gram staining or other stains.<br><br>**Culture, isolation and identification:** clinical material will be evaluated with the microscopy results, directing the seeding procedures in specific media. The growth of microorganisms in the different culture media used provides the first information for their identification. Automated methods generally use the same tests as manual identification and increase the number of tests and can characterize with greater certainty and better discrimination of unusual genera and species.<br><br>**Sensitivity profile:** antimicrobial sensitivity tests are performed according to the CLSI criteria.<br><br>**Disclosure of results of sensitivity profile:** performed through partial and final reports, for decision making by the clinical staff and re-evaluation of the empirical therapy in force.<br><br>**Analysis of changes in the sensitivity profile and investigation of multidrug-resistant microorganisms:** analyses of the frequency of appearance of multidrug-resistant pathogens, as well as identification of resistance mechanisms are shared with the HICC to feed back the management systems of antimicrobial use in the hospital. | |
| **Human Resources***<br>33 clinical pathology technicians<br>5 laboratory analysts<br>3 microbiology analysts<br>3 administrative assistants<br>2 general services assistants<br>1 pathologist<br>1 coordinator | | |

*In the Human Resources section, the total number of professionals in the HICC, HP and DSL departments were highlighted.

## II - Checklist of the essential elements

| **Core elements of hospital antibiotic stewardship programs: assessment tool** | | |
|---|---|---|
| **Hospital Leadership Commitment** | **YES** | **NO** |
| Does the hospital have a formal, written policy of support from senior hospital management in efforts to improve antimicrobial use in the institution? | **X** | |
| Does the policy ensure that staff in departments/sectors have sufficient time to contribute to activities related to antimicrobial use management? | | **X** |
| Are there goals related to the control of antimicrobial use in the Hospital's Strategic Plan? | **X** | |

| | | |
|---|---|---|
| Is there support for the training and continuing education of the hospital's health professionals on topics related to infection control, microbial resistance and management of antimicrobial use? | **X** | |
| Does the institution have a microbiology laboratory? | **X** | |
| Does the microbiology laboratory have automated systems for identification (or Maldi-TOF) or sensitivity testing (fusion disc or other tests that determine the minimum inhibitory concentration - MIC) and agility in providing culture/antibiogram results within 72 hours? | **X** | |
| Does the Hospital have an Antimicrobial Use Management Program? | **X** | |
| Are there human, financial and IT resources necessary to implement the Antimicrobial Use Management Program in the hospital? | **X** | |
| Do the components of the operational team have a specific time frame defined for the execution of the Program's actions? | | **X** |
| **DEFINITION OF RESPONSIBILITY** | **YES** | **NO** |
| Is there a team of professionals responsible for controlling the use of antimicrobials in the hospital? | **X** | |
| If there is an Antimicrobial Stewardship Program in place: | | |
| Has an operational team responsible for designing and implementing the Program been formally appointed? | | **X** |
| Is there an appointed operational team leader who is responsible for the implementation and results of the hospital's Program? | | **X** |
| Is the Program operational leader an infectious disease infectologist, a clinical pharmacist or other professional with expertise in infectious diseases? | | **X** |
| Is there an appointed clinical pharmacist responsible for working towards managing the use of antimicrobials in the hospital? | | **X** |
| Is the Program management team interdisciplinary? | | **X** |
| Indicate the people below who are part of the program management team:<br><br>( ) Clinician ( ) Nurse ( ) Infectologist ( ) Clinical Pharmacist ( ) Microbiologist ( ) CCIH representative ( ) Quality control representative ( ) Microbiology laboratory representative ( ) Pharmacy and Therapeutics Commission representative ( ) IT Technology representative ( ) Others | | **X** |
| **EDUCATION** | **YES** | **NO** |
| Does the hospital have a continuing education program for its professionals aimed at increasing awareness of the use of antimicrobials? | **X** | |
| Does the hospital have an education program for patients and accompanying persons/caregivers on the correct use of antimicrobials? | | **X** |
| **DEVELOPMENT OF ACTIONS TO IMPROVE ANTIMICROBIAL PRESCRIBING** | | |
| **POLICIES** | **YES** | **NO** |
| Does the hospital have protocols for the main clinical syndromes, based on national guidelines and local epidemiological and microbiological profiles, to assist in the selection of antimicrobials for common clinical conditions? | **X** | |
| Are the protocols widely disseminated to all stakeholders and are professionals properly trained in their adoption? | **X** | |
| Does the hospital have an institutional policy that requires prescribers to document the dose, duration, and indication of all prescribed antimicrobials in the prescription? | **X** | |

| **STRATEGIC ACTIONS FOR THE MANAGEMENT OF ANTIMICROBIAL USE** | **YES** | **NO** |
|---|---|---|
| Is there a data collection tool for managing the use of antimicrobials? If yes, which ones? | X | |
| Is there a database for managing the use of antimicrobials? If yes, which? | X | |
| Does a physician or pharmacist review prescriptions for specific antimicrobial agents (i.e. prospective audit) in the hospital? | X | |
| Are prescribers informed about the compliance of their prescriptions after the audit has taken place? | X | |
| Does the hospital have a form-based restriction system for specific antimicrobials? | X | |
| Does the hospital have a pre-authorization system for specific antibiotics? | X | |
| Does the hospital reviewed of planned outpatient parenteral antibiotic therapy (OPAT)? | X | |
| Does the hospital pharmacy perform antimicrobial dose adjustments in cases of organic dysfunction of the patient? | X | |
| Is antimicrobial dose optimization (pharmacokinetics / pharmacodynamics) performed to optimize the treatment of microorganisms with reduced sensitivity? | | X |
| Are there automatic alerts in situations where therapy is unnecessarily duplicated? | X | |
| Does the hospital have automatic time-sensitive stop orders for specific antimicrobial prescriptions, especially antibiotics administered for surgical prophylaxis? | | X |
| Does the hospital perform de-escalation of antimicrobials when indicated? | X | |
| Does the hospital perform culture-guided antimicrobial therapy? | X | |
| Does the hospital perform serum monitoring? If yes, which? | X | |
| Does the hospital perform antimicrobial use management using biomarkers? If yes, which? | | X |
| Does the hospital have protocols for treating the following infections and syndromes? | | |
| Community acquired pneumonia | X | |
| Lower urinary tract infection | X | |
| Skin and soft tissue infection | X | |
| Pyelonephritis | X | |
| Surgical prophylaxis | X | |
| Sepsis | X | |
| Meningitis | X | |
| Empiric treatment for oxacillin-resistant *Staphylococcus aureus* | X | |
| *Clostridium difficile* infection - CDI | X | |
| Laboratory confirmed bloodstream infections | X | |
| **PROGRAM MONITORING PROCESS / USE AND CONSUMPTION INDICATORS** | **YES** | **NO** |
| Does your Antimicrobial Stewardship Program monitor adherence to documentation policies (dose, duration and indication)? | X | |
| Does your Program monitor adherence to the institution's clinical protocols? | X | |
| Does the hospital monitor antimicrobial prescriptions with suspension/revision? | X | |
| The hospital monitors antimicrobial use (consumption) on the units or in the institution by one of the following measures: | | |

| | | |
|---|---|---|
| By the average amount, in grams, of antimicrobials used per patient (Defined Daily Dose - DDD)? | **X** | |
| By the counts of antimicrobial (s) administered to patients per day (Days of Therapy - DOT)? | | **X** |
| By the number of days the patient receives antimicrobial(s) regardless of the number of drugs (Lenght of therapy - LOT)? | | **X** |
| **RESULT INDICATORS / OUTCOME** | **YES** | **NO** |
| Does the hospital track *Clostridium difficile* infection rates? | **X** | |
| Does the hospital monitor costs on antimicrobial consumption? | **X** | |
| **DISSEMINATION OF RESULTS** | **YES** | **NO** |
| Has the updated antimicrobial sensitivity profile been disseminated to prescribers in your hospital? | **X** | |
| Have prescribers received direct and personalized communication on how they can improve their antimicrobial prescribing? | **X** | |
| If the Hospital has an Antimicrobial Stewardship Program | | |
| Do you periodically disseminate the results of the Program to all professionals in the Hospital? | | **X** |
| Does your Program operational team disseminate specific reports on antimicrobial use to prescribers? | | **X** |
| Is information on antimicrobial use and microbial resistance regularly passed to all sectors of the hospital relevant to the Program? | **X** | |
| Have the results, objectives and targets been disseminated to senior hospital management and all sectors involved in the Program? | **X** | |
